# QTL Mapping of Six Spike and Stem Traits in Hybrid Population of *Agropyron* Gaertn. in Multiple Environments

**DOI:** 10.3389/fpls.2018.01422

**Published:** 2018-10-30

**Authors:** Yonghe Che, Nan Song, Yanping Yang, Xinming Yang, Qingqing Duan, Yan Zhang, Yuqing Lu, Xuqing Li, Jinpeng Zhang, Xiuquan Li, Shenghui Zhou, Lihui Li, Weihua Liu

**Affiliations:** ^1^College of Agronomy and Biotechnology, Hebei Normal University of Science and Technology, Qinhuangdao, China; ^2^Institute of Crop Sciences, Chinese Academy of Agricultural Sciences, Beijing, China

**Keywords:** *Agropyron* Gaertn., major QTLs, stable QTLs, spike and stem traits, SNP markers

## Abstract

Most *Agropyron* Gaertn. species are excellent sources of forage. The derivative lines of wheat-*Agropyron cristatum* show elite agronomic traits, and some are valuable for wheat breeding. The species of *Agropyron* Gaertn. was mainly recognized by the spike morphology in traditional taxon. Six traits, including spike length (SL), ear stem length (ESL), the second internodes length (SIL), spikelet number per spike (SNS), floret number per spikelet (FNS), and grain number per spikelet (GNS), are vital to morphology studies and also influences the forage crop yield. To elucidate the genetic basis of spike and stem traits, a quantitative trait locus (QTL) analysis was conducted in a cross-pollinated (CP) hybrid population derived from a cross between two diverse parents, *Agropyron mongolicum* Keng Z2098 and *A. cristatum* (L.) Gaertn. Z1842, evaluated across three ecotopes (Langfang, Changli, and Guyuan of Hebei, China) over 3 years (from 2014 to 2016). Construction of a high-density linkage map was based on 1,023 single-nucleotide polymorphism (SNP) markers, covering 907.8 cM of the whole *Agropyron* genome. A total of 306 QTLs with single QTL in different environments explaining 0.07–33.21% of the phenotypic variation were detected for study traits. Seven major-effect QTLs were identified, including one for ESL on chromosome 3, one for SIL on chromosome 5, three for SL (two on chromosome 2 and one on chromosome 4), and two for SNS on chromosomes 3 and 7. Also, seven stable QTLs, including four for ESL, one for SL, one for GNS, and one for FNS, were mainly mapped on chromosomes 2, 3, 4, 5, and 7, respectively, elucidating 0.25–14.98% of the phenotypic variations. On the use of *Agropyron* CP hybrid population to identify QTL determining spike and stem traits for the first time, these QTLs for six traits would provide a theoretical reference for the molecular marker-assisted selection in the improvement of forage and cereal crop species.

## Introduction

*Agropyron* Gaertn. is an important wild relative of wheat that has the genome of P: diploid, PP, 2*n* = 2*x* = 14; tetraploid, PPPP, 2*n* = 4*x* = 28; and hexaploid, PPPPPP, 2*n* = 6*x* = 42 (Dewey, 1984; Fordlloyd et al., 2011). The most of *Agropyron* species are excellent sources of forage and habitat for livestock and wildlife, and they are also valued for weed control, habitat use, soil stabilization, and watershed management (Wang, 2011). *Agropyron* species possess a lot of useful characteristics, such as the tolerance to drought (Asay and Johnson, 1990) and cold, resistance to diseases (Lu et al., 2015), and high yield traits (Dong et al., 1992). It is a quality forage for grassland improvement and a valuable genetic resource for wheat.

*Agropyron cristatum* (L.) Gaertn. is known as an important model species of *Agropyron*, originating in Iran and distributed in arid, semi-arid, and alpine regions (Dewey, 1984). *A. mongolicum* Keng, a unique species found in China, is mainly distributed in Shaanxi, Ningxia, Inner Mongolia, Gansu, Shanxi, and other areas (Guo, 1987). Both *A. cristatum* and *A. mongolicum* are diploids, but the two are very different in general morphology (Dewey, 1981, 1984). In traditional taxon, the species of *Agropyron* Gaertn. was mainly recognized by the spike morphology. *A. mongolicum* differs from *A. cristatum* in its narrow, linear spikes. All other diploid accessions within genus *Agropyron* were similar to *A. cristatum* in broad spikes. The two species could hybridize easily, and the F_1_ hybrids show a great advantage (Dewey, 1984; Hsiao et al., 1986). *A. desertorum* (Fisch ex Link) Schlut, *A. mongolicum*, and *A. michnoi* Roshev should be offspring species of *A. cristatum* sharing the same basic genome from the counterpart based on SSR analysis (Che et al., 2015).

The spike and stem traits are vital not only to morphology studies but also to yield (Cui et al., 2014; Li et al., 2017; Zhang et al., 2017). Some excellent genes associated with spike characteristics were found on chromosome 6P of *Agropyron* (Wu et al., 2006; Luan et al., 2010). Spike-related traits are complex quantitative traits controlled by multiple genes, and showed continuous variation in segregating offspring (Kobayashi et al., 2003; Fan et al., 2015). Because each QTL contributes less to phenotype and influenced easily by the environments, it is difficult to study by traditional cytogenetics and quantitative inheritance. QTL mapping provides the possibility of studying complex traits.

QTL analysis for spike-related traits has been studied using different mapping populations. A number of QTLs for spike-related traits have been found in the F_2_, RIL, and BC population in wheat (Huang et al., 2004; Ma et al., 2007; Deng et al., 2011; Jia et al., 2013; Cui et al., 2014). Some QTLs for spike-related traits were also detected in the double haploid (DH) population in rice (Bao et al., 2002; Qiu et al., 2011). *Agropyron* Gaertn., a perennial cross-pollination plant, have characteristics of complex genetic background, long generation cycle, highly heterozygous genome, and self-incompatibility or low self-sufficiency rate (Che et al., 2015; Zhang et al., 2015). Therefore, it is not suitable for isolated populations crossed from homozygous lines and is suitable for the F_1_ population. A new mapping method called “two-way pseudo-testcross” was put forward, providing the possibility to construct a genetic map in heterotic species (Grattapaglia and Sederoff, 1994). The genetic map of *Lolium perenne* in CP (cross pollinated) has been constructed using “Pseudo-testcross,” and QTL associated with stem rust resistance was mapped (Pfender et al., 2011). It was also reported that the genetic map of *Camellia sinensis* was constructed in the CP hybrid population deriving from the heterozygous diploid parents (Hu et al., 2013).

Meanwhile, the P genome of *Agropyron* has not been sequenced because of its huge genome and sequencing cost so far (Zhang et al., 2015; Absattar et al., 2018; Zhou et al., 2018). SLAF-seq (specific-locus amplified fragment sequencing) is an effective way for high-throughput simplified genome sequencing and large-scale development of single-nucleotide polymorphism (SNP) markers and genotyping (Sun et al., 2013). Based on the genetic map of SNP markers constructed by SLAF-seq, QTL analysis for spike and stem traits was carried out to determine the number, effect, and position of QTLs on the chromosomes. It would provide a theoretical basis for molecular marker-assisted selection (MAS) in the future study.

## Materials and methods

### The progress of constructing the F_1_ population

The CP hybrid population used for QTL mapping derived from the interspecific cross of diploid *A. mongolicum* Z2098 (2*n* = 2*x* = 14, PP, female) and *A. cristatum* Z1842 (2*n* = 2*x* = 14, PP, male). The experiment with hybrids of *Agropyron* was carried out in May of 2012 in Langfang of Hebei province. A total of 19 spikes from one single plant of the *A. mongolicum* Z2098 population as the maternal plant were emasculated and isolated by paper bags. Pollen was collected from one single plant of the *A. cristatum* Z1842 population and hybridized with maternal plant through shaking spike. The 174 F_1_ seeds were placed in an incubator at 25°C for germination after they were dry, 135 of which germinated. Then 135 individuals of progeny were planted in the greenhouse in Chinese Academy of Agricultural Sciences, Beijing, China, after which they were clonally propagated from tillers and transplanted 115 ideal seedlings (including two parents) to Langfang (116°70′ E, 39°53′ N), Changli (119°15′ E, 39°72′ N), and Guyuan (115°68′ E, 41°68′ N) of Hebei province in April 2013, April 2014 and September 2014, respectively. A total of 115 individuals (including two parents) were planted in each ecotope, with a spacing of 40 cm between plants and 60 cm between rows. Each material was designed with three replications in each experiment site and managed conventionally.

### Trait measurements

All individuals in each experiment site were selected for the measurement of six traits, including spike length (SL), ear stem length (ESL), the second internodes length (SIL), spikelet number per spike (SNS), floret number per spikelet (FNS), and grain number per spikelet (GNS) in 2015 and 2016 in three environments. Five of all study traits except ESL were surveyed in 2014 in Langfang. Ten effective tillers randomly sampled after maturing from each plant were surveyed for an average of study trait of an individual. The traits were measured as previously described (Li and Li, 2006).

### Statistical analysis

The phenotypic data and the correlation coefficients between pairs of all six traits of the CP hybrid population were analyzed using SPSS 20.0 software (SPSS Inc., Chicago, IL, USA).

### QTL analysis

A population of 113 individuals of the hybrid between two species, *A. mongolicum* and *A. crystatum*, was used as the mapping population, and a threshold range 3–10 of independence LOD was applied to group and construct the high-density molecular map. The genetic map of the *Agropyron* whole genome based on the SLAF-seq technique was constructed and distributed on seven linkage groups (Zhang et al., 2015). The final map consisting of 1,023 SNP markers spanned a total of 907.8 cM (centi-morgan), with an average distance of 1.5 cM between markers. The number of markers was 115–225 per linkage group, with an average of 146 markers per linkage group.

The phenotypic data, an average of each trait from three replicates in each environment, were examined by the Kruskal-Wallis test, and the data satisfying normal distribution were used for the detection of QTL. QTL analysis was performed using inclusive composite interval mapping in the GACD software (Li et al., 2008). An LOD score of 2.5 was set as a threshold, and a walking speed for all QTLs was 1.0 cM and the PIN value was 0.001 (Wang, 2009). A QTL with an LOD value >2.5 and a contribution rate of >10% detected in different environments was defined as a major QTL, and the QTL detected in at least three different environments was regarded as a stable QTL (Cui et al., 2011, Raihan et al., 2016; Fan et al., 2017). QTLs for the same traits detected in different environments were considered to be the same if the confidence intervals overlapped. The QTL was named “q + the first letter of the place name (C represents Changli, L represents Langfang, G represents Guyuan) + the abbreviation of trait name + year (1, 2, 3 represents the year of 2014, 2015, 2016, respectively) + chromosome + serial number” (Mccouch et al., 1997).

## Results

### Phenotypic variation and correlation between traits

**Figure 1:**
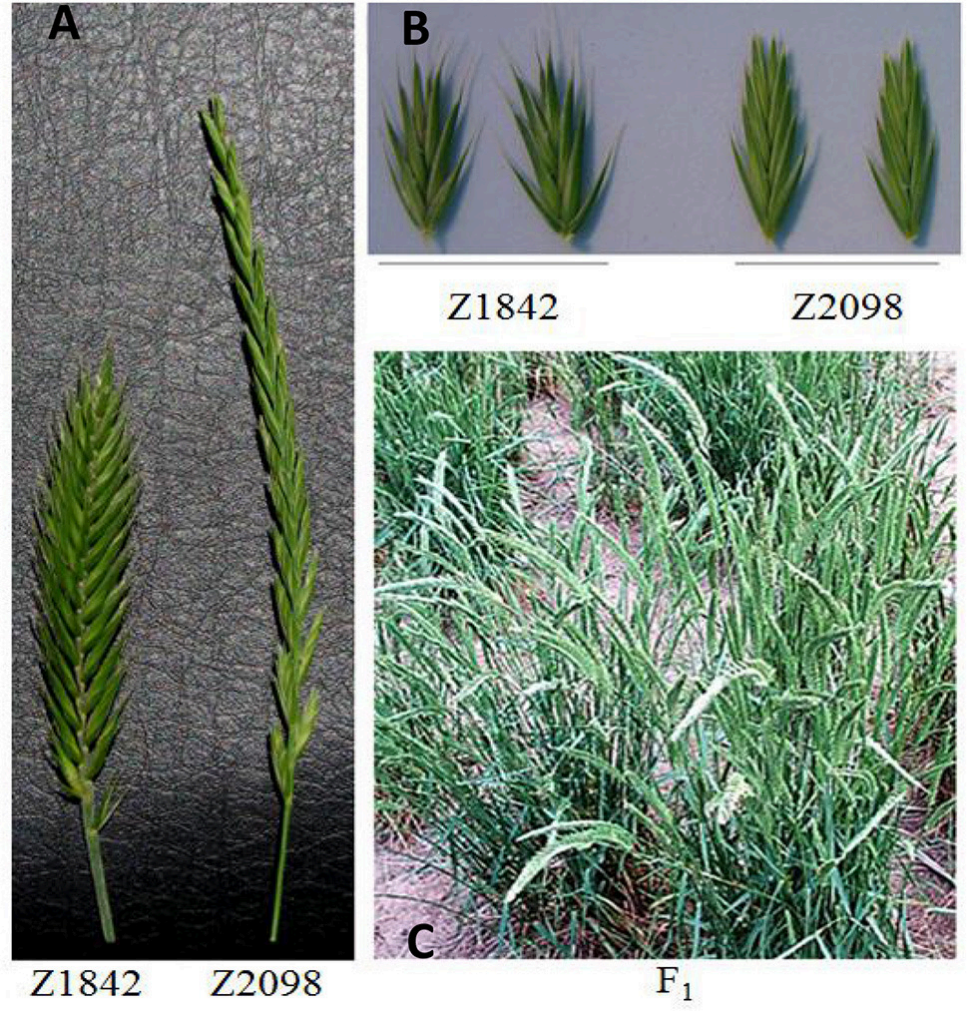
Comparison of the morphology of the parents **(A,B)** and the F_1_ hybrid plants of Z2098/Z1842 **(C)**.

The parents and CP hybrid population of *Agropyron* had a significant difference in spike morphology (Figure 1). *A. cristatum* had a board and short spike, whereas *A. mongolicum* had a narrow and long spike. F_1_ hybrid plants showed abundant morphologic diversity, spikes of some plant from which were longer than that of *A. cristatum* and border than *A. mongolicum*, respectively. There were abundant diversity tillers even in an individual.

Six traits of *Agropyron* in three ecotopes in 3 years are presented in Table 1. The parents showed a significant variation in three different environments, and the average of CP hybrid population for most of traits was closer to the female than the male (Table 1; Supplementary Material Figures A–C). Similarly, spike and stem traits also showed a great range of variation in the CP hybrid population, such as the minimum for ESL was 24.70 cm, maximum up to 90.03 cm in three ecotopes in 3 years. The spike-related traits all had an obvious difference among years (Supplementary Material Figures A–C).

**Table 1:** Summary of agronomic traits of *Agropyron* in three environments in 3 years.

**Environment**	**Trait**	**Year**	***Mongolicum* Z2098**	***Cristatum* Z1842**	**CP population**				
					**Mean ±SD**	**Coefficient of variation**	**Min–Max**	**Skewness**	**Kurtosis**
Langfang	ESL	2014	35.20	27.33	37.28 ± 4.20	11.26	27.44–46.52	0.01	−0.61
		2015	36.04	26.17	38.46 ± 4.48	11.65	24.70–50.80	−0.15	0.76
		2016	30.41	28.48	47.86 ± 7.16	14.96	32.00–65.00	3.32	19.35
	SIL	2015	6.81	7.68	13.58 ± 3.07	22.59	8.45–21.65	0.55	−0.19
		2016	6.00	10.22	17.27 ± 10.20	59.07	0.00–35.00	−0.30	−0.47
	SL	2014	9.19	9.19	8.08 ± 0.96	11.85	4.80–10.84	−0.04	1.21
		2015	10.09	9.95	8.39 ± 1.08	12.82	6.12–10.89	0.18	−0.56
		2016	8.43	7.30	6.59 ± 1.50	22.81	3.40–10.30	0.67	0.84
	SNS	2014	27.30	25.70	27.45 ± 3.74	13.61	12.60–35.75	−0.11	1.61
		2015	34.40	26.10	31.84 ± 3.49	10.94	23.50–41.50	0.12	0.19
		2016	35.00	25.30	27.57 ± 6.80	24.68	16.00–46.00	0.13	0.94
	FNS	2014	7.60	4.85	7.37 ± 0.98	13.31	4.67–10.10	0.38	0.37
		2015	5.30	5.70	5.94 ± 1.01	17.04	4.33–11.56	1.74	7.62
		2016	7.50	4.00	7.79 ± 2.89	37.12	4.00–22.00	0.23	−0.42
	GNS	2014	1.10	1.55	1.24 ± 0.37	30.56	0.30–2.13	0.10	0.04
		2015	1.30	2.00	1.46 ± 0.56	38.43	0.40–3.00	0.28	−0.31
		2016	1.90	1.10	1.38 ± 0.71	51.45	0.00–8.00	−0.19	0.12
Changli	ESL	2015	44.48	30.60	40.44 ± 5.72	14.15	4.60–51.44	−2.20	12.93
		2016	51.03	47.59	48.55 ± 5.91	12.17	32.77–74.72	0.46	3.04
	SIL	2015	9.08	13.27	14.06 ± 2.97	21.11	7.26–25.90	0.67	1.54
		2016	16.60	15.23	15.05 ± 3.95	26.26	0.00–22.87	−0.92	2.04
	SL	2015	9.89	8.92	7.48 ± 0.62	8.24	5.94–10.84	0.12	0.26
		2016	8.73	8.63	8.49 ± 1.90	22.37	6.29–25.92	7.27	66.49
	SNS	2015	28.40	24.00	31.75 ± 3.33	10.49	24.50–39.10	−0.27	0.40
		2016	26.33	27.53	29.46 ± 5.70	19.36	18.80–40.27	4.84	38.29
	FNS	2015	8.00	6.90	6.23 ± 1.25	20.10	4.60–12.70	2.55	11.52
		2016	8.93	6.06	9.02 ± 3.15	34.86	5.60–35.80	6.63	52.35
	GNS	2015	1.20	1.20	1.92 ± 0.69	35.66	0.80–4.10	0.72	0.48
		2016	1.47	1.47	1.23 ± 0.48	38.79	0.00–2.53	0.55	0.34
Guyuan	ESL	2015	34.87	33.81	43.77 ± 5.01	11.45	32.22–54.00	−0.10	0.51
		2016	40.72	44.00	42.93.±7.26	16.90	30.61–90.03	−0.07	−0.40
	SIL	2015	18.47	18.10	18.12 ± 3.36	18.55	11.66–27.03	0.47	−0.14
		2016	17.54	15.20	12.51.±4.00	31.96	2.55–20.27	−0.70	−0.64
	SL	2015	8.50	5.08	7.48 ± 0.62	8.24	5.64–8.85	0.12	0.26
		2016	8.00	7.64	8.32 ± 1.00	12.01	6.18–11.76	0.14	−0.38
	SNS	2015	18.00	18.25	23.19 ± 2.46	10.61	18.63–29.50	0.36	−0.38
		2016	30.67	24.00	33.88 ± 2.96	8.73	26.10–42.80	0.59	−0.10
	FNS	2015	11.70	11.70	10.56 ± 1.37	12.93	7.90–15.00	0.52	0.31
		2016	8.22	4.00	5.06 ± 0.74	14.70	3.40–6.90	1.46	5.04
	GNS	2015	1.10	1.10	1.38 ± 0.83	60.03	0.00–4.33	1.00	2.04
		2016	1.69	1.00	1.03 ± 0.41	39.97	0.10–2.10	2.99	10.72

There were certain differences in every trait among different environments. For the CP hybrid population, the mean values of six traits in Guyuan and Changli were much higher than Langfang. For instance, the mean for ESL in Changli and Guyuan was 40.44 and 43.77 cm in 2015, respectively, higher than that of Langfang (38.46 cm). For most of traits, there was also a big difference in the same trait among different years. In 3 years, most of traits in Langfang in 2015 showed larger than other 2 years. For example, the mean value of SL was 8.08, 8.39, and 6.59 cm, respectively, in three consecutive years in Langfang. It indicated that Guyuan and Changli were more suitable for *Agropyron* planting. The coefficient of variation in six traits ranged from 8.24 to 60.03%. For most traits, there was no significant difference in the coefficient of variation in three environments in different years with exception of FNS, SIL, and SNS in Guyuan. The coefficient of variation for SL showed a significant difference in 2015 (Changli, 8.24; Guyuan, 8.24) and 2016 (Changli, 22.37; Guyuan, 22.81).

The correlation coefficients between study traits are shown in Table 2. There were differences in the correlation between traits. There was a positive correlation for all traits between 2015 and 2016 in Langfang and Changli (*P* < 0.01). However, there was a negative correlation between ESL, SIL and GNS in Guyuan (*r* = −0.02, −0.01, and −0.03, respectively; *P* < 0.05). In 2015, SIL was significantly positively correlated with ESL (*r* = 0.37, *P* < 0.01) and SNS in Langfang. Moreover, SIL was negatively related to SL, GNS, and FNS (*P* < 0.05, *r* = −0.13, −0.04, and −0.08). There was a significantly or highly significantly positive correlation between SL and FNS in three ecotopes in 2015, and SL was also significantly positively correlated with FNS in Langfang (*r* = 0.25, *P* < 0.01) and had a positive correlation in Changli and Guyuan (*r* = 0.04 and 0.13, *P* < 0.05) in 2016. There was a negative correlation or significantly negative correlation between SNS and GNS in 2015 in three ecotopes. These results suggested that study trait values of CP hybrid population were rich in variation at different environments and were feasible for QTL analysis.

**Table 2:** Correlation coefficients for agronomic traits in the *Agropyron* CP hybrid population.

**Environment**	**Trait**	**ESL**	**SIL**	**SL**	**SNS**	**GNS**	**FNS**
Langfang	ESL	**0.20**	−0.06	−0.01	0.10	0.00	−0.07
	SIL	0.37^**^	**0.19**	0.12	0.07	0.01	0.03
	SL	0.11	−0.13	0.39^**^	0.33^**^	0.15	0.25^*^
	SNS	−0.05	0.23^*^	0.57^**^	0.47^**^	0.15	0.09
	GNS	0.15	−0.04	−0.04	−0.05	**0.04**	−0.07
	FNS	0.09	−0.08	0.47^**^	−0.19	0.26^**^	**0.10**
Changli	ESL	**0.18**	0.27^**^	0.23^*^	0.19^*^	0.10	0.04
	SIL	−0.18	0.32^**^	0.05	−0.05	0.19^*^	0.17
	SL	0.12	0.06	**0.11**	0.11	0.04	0.04
	SNS	−0.10	−0.15	0.60^**^	**0.13**	0.05	0.08
	GNS	0.19	0.09	0.34^**^	−0.28^**^	0.41^**^	−0.08
	FNS	0.02	0.04	0.24^*^	−0.01	0.13	**0.13**
Guyuan	ESL	−**0.02**	−0.13	0.03	0.04	−0.06	0.01
	SIL	0.01	−**0.01**	0.05	0.05	0.10	0.11
	SL	−0.02	0.09	**0.16**	0.63^**^	0.11	0.13
	SNS	0.10	−0.18	0.45^**^	**0.14**	0.05	0.07
	GNS	0.15	−0.21	0.24^*^	−0.30^**^	−**0.03**	0.54^**^
	FNS	0.24^*^	−0.09	0.30^**^	−0.13	−0.17	−**0.06**

Lower and upper diagonal represent in year of 2015 and 2016; Bold font represents the correlation between different years, respectively; ^*^ and ^**^ represent significant differences at the 5 and 1% level, respectively.

### QTL analysis for study traits

A total 306 QTLs for six study traits were detected on seven linkage groups in three ecotopes in 3 years, with single QTL explaining 0.07% to 33.21% of the phenotypic variation. There were 77 QTLs for ESL, 35 QTLs for SIL, 51 QTLs for SL, 28 QTLs for SNS, 47 QTLs for FNS, and 68 QTLs for GNS, respectively. Seven major QTLs and seven stable QTLs were detected on chromosomes 2, 3, 4, 5, and 7.

### Ear stem length

**Figure 2:**
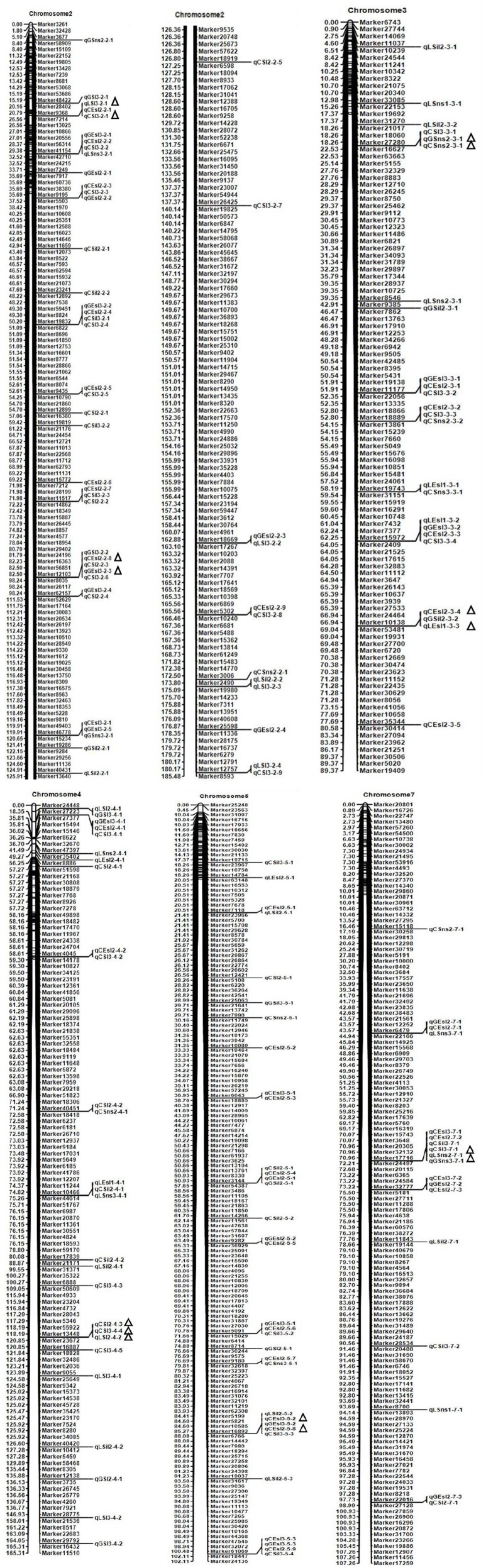
Chromosome locations of some QTLs and major QTLs for six spike and stem traits detected in the *Agropyron* CP hybrid population. Δ represents the location of the major QTL detected in three environments.

Seventy-seven QTLs for ESL were detected. The major QTL (*qCEsl2-3-4, qLEsl1-3-3*) explaining totally 16.18% of the phenotypic variations was mapped at position 67–68 cM on chromosomes 3 flanking the marker interval Marker10138–Marker53481 (Table 3; Figure 2). The additive effect of *qCEsl2-3-4* from female was positive, showing that there was a big contribution from female, whereas there was a negative additive effect of *qLEsl1-3-3* from male and female. Four stable QTLs (*qLEsl1-3-2, qCEsl2-3-3, qGEsl3-3-2*; *qCEsl2-5-8, qCEsl3-5-2, qGEsl3-5-2*; *qCEsl2-5-9, qCEsl3-5-3, qGEsl3-5-3*; *qCEsl2-7-3, qCEsl3-7-2, qGEsl2-7-2*) were found in at least three environments of 3 years (Table 4).

**Table 3:** Summary of major QTLs detected in *Agropyron* Gaertn.

**Trait**	**Environment**	**Year**	**QTL**	**Chr**.	**Site**	**Marker interval**	**LOD**	**phenotypic variation explained (%)**	**Additive effect (female)**	**Additive effect (male)**
ESL	Changli	2015	qCEsl2-3-4	3	67	Marker10138–Marker53481	8.39	1.00	−10.29	11.26
	Langfang	2014	qLEsl1-3-3	3	68	Marker10138–Marker53481	2.75	15.18	−0.62	−0.15
SIL	Langfang	2015	qLSil2-5-2	5	87	Marker16892–Marker6766	2.55	11.49	−0.16	0.52
	Changli	2016	qCSil3-5-3	5	88	Marker16892–Marker6766	3.31	3.93	−4.15	4.25
SL	Langfang	2016	qLSl3-2-1	2	25	Marker9368–Marker7214	2.63	12.76	0.34	0.44
	Changli	2016	qCSl3-2-1	2	26	Marker9368–Marker7214	7.27	0.25	−2.17	2.00
	Guyuan	2016	qGSl3-2-2	2	95	Marker12103–Marker8035	2.93	11.35	0.13	−0.27
	Changli	2015	qCSl2-2-3	2	96	Marker12103–Marker8035	3.73	1.30	0.37	−0.49
	Changli	2015	qCSl2-4-3	4	119	Marker13448–Marker23672	3.27	1.39	0.39	−0.43
	Changli	2016	qCSl3-4-4	4	119	Marker13448–Marker23672	4.67	0.25	−2.04	2.00
	Langfang	2015	qLSl2-4-2	4	120	Marker13448–Marker23672	2.79	14.98	0.18	−0.41
SNS	Changli	2015	qCSns2-3-1	3	19	Marker27280–Marker16627	5.69	18.68	−0.68	−0.02
	Guyuan	2015	qGSns2-3-1	3	19	Marker27280–Marker16627	2.98	11.17	−0.81	0.12
	Guyuan	2016	qGSns3-7-1	7	71	Marker17716–Marker24497	5.81	5.90	4.11	−3.36
	Langfang	2015	qLSns2-7-1	7	71	Marker17716–Marker24497	5.32	14.11	−1.69	2.89

**Table 4:** Summary of stable QTLs detected in *Agropyron* Gaertn.

**Trait**	**Environment**	**Year**	**QTL**	**Chr**.	**Site**	**Marker interval**	**LOD**	**phenotypic variation explained (%)**	**Additive effect (female)**	**Additive effect (male)**
ESL	Langfang	2014	qLEsl1-3-2	3	63	Marker15972–Marker2409	2.52	9.28	−0.73	−0.08
	Changli	2015	qCEsl2-3-3	3	64	Marker15972–Marker2409	5.29	2.16	−8.81	8.50
	Guyuan	2016	qGEsl3-3-2	3	64	Marker15972–Marker2409	10.70	0.89	−8.74	9.07
	Changli	2015	qCEsl2-5-8	5	88	Marker16892–Marker6766	4.88	1.47	4.07	−3.29
	Changli	2016	qCEsl3-5-2	5	88	Marker16892–Marker6766	6.93	2.24	8.41	−8.45
	Guyuan	2016	qGEsl3-5-2	5	88	Marker16892–Marker6766	11.07	0.98	−10.96	11.39
	Changli	2015	qCEsl2-5-9	5	102	Marker61069–Marker18447	6.60	1.78	9.57	−8.20
	Changli	2016	qCEsl3-5-3	5	102	Marker61069–Marker18447	6.78	1.95	5.80	−5.52
	Guyuan	2016	qGEsl3-5-3	5	102	Marker61069–Marker18447	11.25	1.00	−7.40	8.16
	Changli	2015	qCEsl2-7-3	7	74	Marker32777–Marker5181	2.76	1.16	−6.24	6.81
	Changli	2016	qCEsl3-7-2	7	74	Marker32777–Marker5181	3.61	6.71	1.57	−2.57
	Guyuan	2015	qGEsl2-7-2	7	74	Marker32777–Marker5181	9.11	0.90	8.82	−9.09
SL	Changli	2015	qCSl2-4-3	4	119	Marker13448–Marker23672	3.27	1.39	0.39	−0.43
	Changli	2016	qCSl3-4-4	4	119	Marker13448–Marker23672	4.67	0.25	−2.04	2.00
	Langfang	2015	qLSl2-4-2	4	120	Marker13448–Marker23672	2.79	14.98	0.18	−0.41
GNS	Langfang	2016	qLGns3-2-1	2	25	Marker9368–Marker7214	8.60	0.94	0.66	−0.72
	Langfang	2014	qLGns1-2-1	2	26	Marker9368–Marker7214	2.69	9.18	−0.03	0.06
	Guyuan	2015	qGGns2-2-1	2	26	Marker9368–Marker7214	2.78	1.24	−0.84	0.77
FNS	Changli	2016	qCFns3-5-1	5	27	Marker12421–Marker5108	3.01	5.35	−0.60	0.48
	Changli	2015	qCFns2-5-1	5	27	Marker12421–Marker5108	14.55	0.74	−1.19	1.23
	Guyuan	2015	qGFns2-5-1	5	27	Marker12421–Marker5108	4.27	9.84	−0.81	1.05

### The second internodes length

For SIL, there were 35 QTLs, 11, 6, and 18 QTLs in Langfang, Guyuan, and Changli, respectively. The major QTL of *qLSil2-5-2, qCSil3-5-3* with LOD values of 2.55 and 3.31 was identified at position 87–88 cM on chromosome 5 flanking the marker interval Marker16892–Marker6766, and explained a total of 15.42% of the phenotypic variations (Table 3; Figure 2). The QTL of *qLSil2-5-2* and *qCSil3-5-3* showed a positive additive effect from female.

### Spike length

There were 51 QTLs for SL, including three major QTLs and one stable QTL. The major QT (*qLSl3-2-1, qLSl3-2-1*) was located at position 25–26 cM on chromosome 2 flanking the markers Marker9368 and Marker7214, explaining totally 13.01% of the phenotypic variations. One major QTL (*qGSl3-2-2, qCSl2-2-3*) explaining a total of 12.65% of the phenotypic variations was located on chromosome 2 bound by the marker interval Marker12103–Marker8035 at 95–96 cM and had a negative additive effect from female. Another major QTL (*qCSl2-4-3, qCSl3-4-4, qLSl2-4-2*) with 16.62% of the phenotypic variations was mapped at position 119–120 cM on chromosome 4 flanking the marker interval Marker13448–Marker23672 and showed a negative additive effect, and the major QTL was also a stable QTL that was detected in three environments (Tables 3, 4; Figure 2).

### Spikelet number per spike

Twenty-eight QTLs for SNS including two major QTLs were detected. A major QTL (*qCSns2-3-1, qGSns2-3-1*) flanking the marker interval Marker27280–Marker16627 was revealed in multiple environments in 2015, which was detected at 19 cM on chromosome 3, and explained totally 29.85% of the phenotypic variations (Table 3; Figure 2). The major QTL showed a positive additive effect. Another major QTL (*qGSns3-7-1, qGSns3-7-1*) explaining 20.01% of the phenotypic variations was mapped at 71 cM on chromosome 7 flanking Marker17716 and Marker24497, and had a positive additive effect. The phenotypic variation for SNS ranged from 2.50 to 18.68%. Fifteen QTLs had positive additive effects and thirteen QTLs had negative additive effects.

### Grain number per spikelet

A total of 68 QTLs for GNS containing one stable QTL were detected. These QTLs were distributed on all chromosomes, with 12, 15, 5, 9, 7, 11, and 9, respectively, on chromosome 1 to 7. Most of QTLs had a negative additive effect from female. The stable QTL (*qLGns3-2-1, qLGns1-2-1, qGGns2-2-1*) was detected in Langfang in 2016 and Langfang, Guyuan in 2014, and was mapped at position 25–26 cM on chromosome 2 flanking Marker9368 and Marker7214 (Table 4).

### Flower number per spikelet

Forty-seven QTLs were detected on all seven chromosomes in three ecotopes in 3 years. A stable QTL (*qCFns3-5-1, qCFns2-5-1, qGns2-5-1*) located at 27 cM on chromosome 5 flanking Marker12421 and Marker5108 was detected in Changli in 2015 and 2016 and Guyuan in 2016 (Table 4). A half of QTLs had a positive additive effect, 17 of which were from female. The QTLs for FNS mapped at 6 cM on chromosome 7 were identified in Guyuan for 2 consecutive years, having a negative additive effect from male.

### Distribution of QTL

QTL for every trait was detected in every chromosome. The number of QTLs ranged from 28 to 77. For ESL, 77 QTLs were detected, which was the highest number of QTLs in all traits, whereas the number of QTLs for SNS was lowest, that is, 28. There were 40, 67, 40, 38, 56, 31, and 34 QTLs from chromosome 1 to 7, respectively. The highest number of QTLs (67) were detected on chromosome 2, whereas there were the lowest number of QTLs (31) detected on chromosome 6. There were many QTLs that controlled multiple traits at the same time in every chromosome. There were QTLs that controlled different traits at the same locus, which was generally considered to be “pleiotropism.” For example, the QTLs controlling all six traits were detected in the Marker17716–Marker24497 interval on chromosome 7.

### The major QTL for study traits

There were seven major QTLs (Table 3; Figure 2), including one for ESL, one for SIL, three for SL, and two for SNS. These major QTLs were mainly distributed on chromosomes 2, 3, 4, 5, and 7. All these major QTLs were detected in two ecotopes, half of which were found in Langfang and Changli, two in Changli and Guyuan, and one was found in Guyuan and Langfang. A few of these QTLs showed a positive additive effect from female, and others exhibited a negative additive effect.

### The stable QTL for study traits

Seven stable QTLs, including four for ESL, two on chromosome 5, one on chromosome 3, and one on chromosome 7; one for SL on chromosome 4; one for GNS on chromosome 2; and one for FNS on chromosome 5, were detected in at least three environments (Table 4). These stable QTLs were mainly mapped on chromosomes 2, 3, 4, 5, and 7, elucidating the phenotypic variation of 0.25% to 14.98%. Most of stable QTLs had a negative additive effect, a few of which were from male.

## Discussion

### The effect of environments on phenotypic traits and QTLs

The climate and soil in the three research regions is obviously different; Guyuan belongs to a high-altitude grassland climate and Changli is more humid all year than Langfang. Therefore, the growth pattern of *Agropyron* is also different. The ESL, SIL, and FNS of the CP hybrid population of *Agropyron* showed an increasing trend in Langfang from 2015 to 2016; however, the SL, SNS, and GNS showed a downward trend. The coefficient of variation of the CP hybrid population varied greatly from region to region, especially to Guyuan. There were also significant differences for spike and stem traits among years. The correlation between most traits was consistent with different environments or with different years, but the natural environment also motivated the correlation between the various traits.

The number of QTLs detected for the same traits varies in different ecotopes. Nineteen QTLs for ESL were detected in Changli in 2015, whereas there were only eight QTLs in Guyuan and Langfang in 2015. Similarly, there were different numbers of QTLs detected in different years. There were four QTLs, eight QTLs, and one QTL for ESL in Langfang in 2014, 2015, and 2016, respectively. This showed that the environment had a significant effect on the phenotypic traits of *Agropyron*.

The CP hybrid population of *Agropyron* was constructed and used as a mapping population for QTL analysis for the first time. Spike and stem traits are classical quantitative traits controlled by many genes. The QTLs detected in more than one environment were more accurate and reliable than the QTLs detected in a single environment (Paterson et al., 1991; Veldboom and Lee, 1996). Seven major QTLs existed stably in at least two environments in this study. Therefore, the results could provide a theoretical reference for understanding the genome structure of *Agropyron*. The quantitative traits would have deepened understanding through new analytical models and population designs based on the present study in the future.

The QTLs of *qGEsl2-2-1* and *qGEsl2-2-2* were detected in Guyuan in 2015 and had an adjacent location (≤2 cM); it may be the same QTL. The QTLs of *qCEsl2-2-3* and *qGEsl2-2-2* had the same confidence interval and were detected at the same site. This indicated that the QTL of *qGEsl2-2-1, qCEsl2-2-3*, and *qGEsl2-2-2* may be a stable QTL. Similarly, The QTL of *qCGns2-6-1, qLGns3-6-4*, and *qGGns2-6-4* found at position 71–72 cM on chromosome 3 could be regarded as a stable QTL; the situation also applied to the QTL of *qGGns2-7-1, qCGns2-7-3*, and *qLGns3-7-3*.

### Pleiotropy and multigenic effect in QTL

The associated traits are often regulated by the same QTL or closely linked QTLs (Paterson et al., 1991; Kato et al., 2000). There were some QTLs for controlling different traits at the same or similar loci, and there was a high correlation among these traits in this study. The QTLs for ESL and SIL were detected on chromosome 5 flanking Marker16892 and Marker6766. The ESL had a highly significantly positive correlation with SIL in Changli in 2016. Likewise, there were QTLs for SL and SNS at 72 cM on chromosome 4. This indicated that there may be multiple tightly linked genes in one locus. There are some reasons for this phenomenon. The genes controlling different traits may be closely linked, or the same gene may affect the performance of different traits. The QTL for ESL had a positive additive effect from male, whereas the QTL for SIL had a positive additive effect from female at the same site on chromosome 5 (Marker16892–Marker6766).

A number of traits governed by QTL can be modified simultaneously by molecular MAS. Meanwhile, there was a disadvantage that if QTLs controlling different traits had an opposite effect, it would be a negative effect on plant breeding. So it is necessary to break the chain through fine mapping. On the contrary, if the QTLs for different traits had an identical positive effect, these QTLs would play an important role in the genetic improvement of crop yield-related traits.

### Contrast with wheat in QTL

The QTLs for SL, SNS, and GNS were located on the same locus (Marker17716–Marker24497) at 71 cM on chromosome 7P in this study. The tight QTL clusters in specific regions of chromosome were in agreement with the phenotypic correlations in these traits. The SL had a highly significantly positive correlation with SNS, and SNS had a highly significantly negative correlation in most instances. Similarly, the QTLs controlling SL and SNS were detected in the same locus on chromosome 7D in wheat, and they are significantly correlated (Ma et al., 2007), and the QTLs for SNS and GNS were found at the same locus of chromosome 7D in wheat (Li et al., 2007). The QTLs for SL and SNS were mapped at 42 cM on chromosome 4P in the present study. Likewise, SL, GNS, and SNS were mapped on the same site on chromosome 4B (Deng et al., 2011). Wheat and *Agropyron* may have homology in this segment and further research is needed. These loci should be focused on in future.

### The importance of part chromosomes

The number of QTLs for GNS on chromosomes 1 and 2 was greater than on other chromosomes. The grain number per spikelet is the most important factor in improving yield. Chromosome 1 and chromosome 2 should be focused on the future research for GNS. Meanwhile, there were 6, 6, 1, 2, 5, and 11 for SL, ESL, SIL, SNS, FNS, and GNS, respectively, on chromosome 6 in the present study. In addition, some research studies have found that certain segments of *Agropyron* chromosome 6P may contain key genes influencing spike characteristics (Wu et al., 2006; Dai and Gao, 2016). These studies have good agreement with the present results.

## Conclusions

We used the *Agropyron* CP hybrid population derived from Z2098/Z1842 to identify QTL determining spike and stem traits across three ecotopes in 3 years for the first time. A total of 306 QTLs were detected on 7 linkage groups across all of the environments in the 3 consecutive years. Seven major QTLs and seven stable QTLs were identified in multiple environments in this study. The effect of additive for QTL was detected, and most of QTLs showed a low additive effect. These works would provide good suggestions for MAS in the improvement of forage and cereal crop species. Fine mapping could be constructed for subsequent research to provide a theoretical basis for the discovery of elite gene.

## Author contributions

YC, WL, and LL designed the research. YC, NS, QD, XuL, and YY performed the research. YC and NS wrote the paper. XY, YZ, JZ, YL, XiL, and SZ participated in the preparation of the reagents and materials in this study.

### Conflict of interest statement

The authors declare that the research was conducted in the absence of any commercial or financial relationships that could be construed as a potential conflict of interest.
