# Supplementary material for: QTL Mapping of Six Spike and Stem Traits in Hybrid Population of Agropyron Gaertn. in Multiple Environments
Source: Front Plant Sci. 2018 Oct 30;9:1422. doi: 10.3389/fpls.2018.01422 (PMC6218563; doi:10.3389/fpls.2018.01422)
Supplement: Supplementary file 1 [file Image_1.pdf]

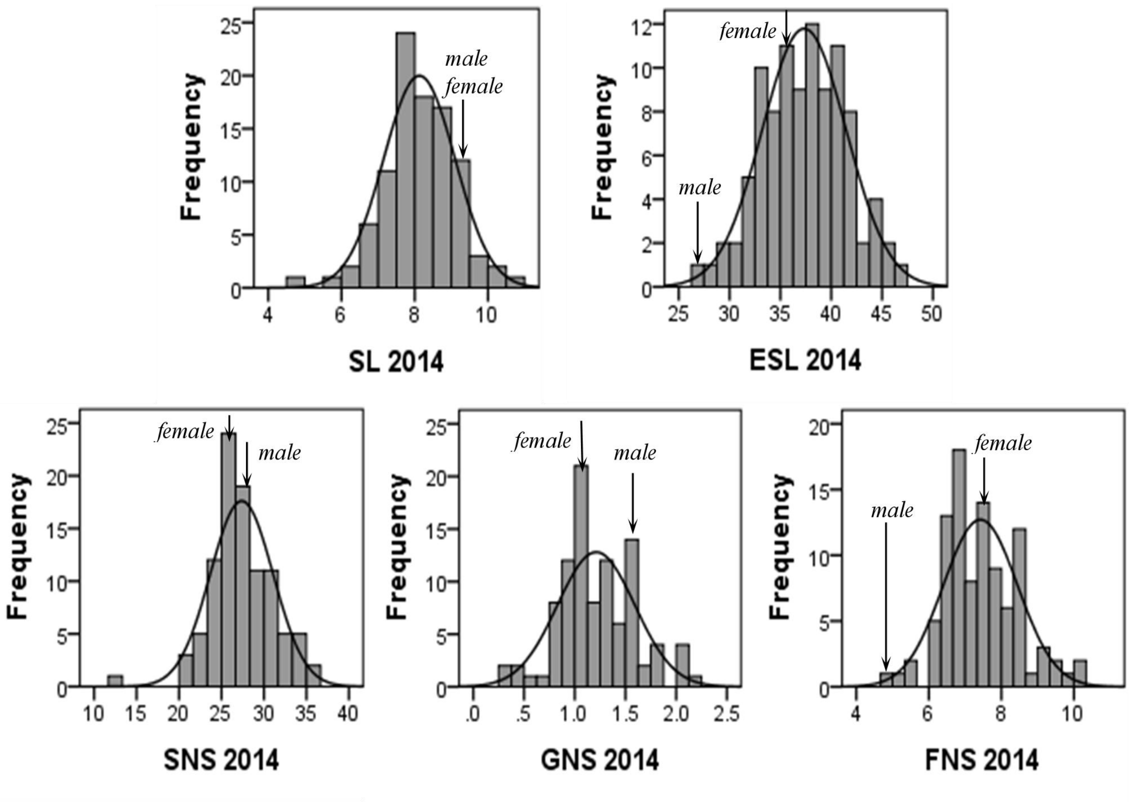

FIGURE A Phenotypic analysis of five traits in Langfang in 2014.

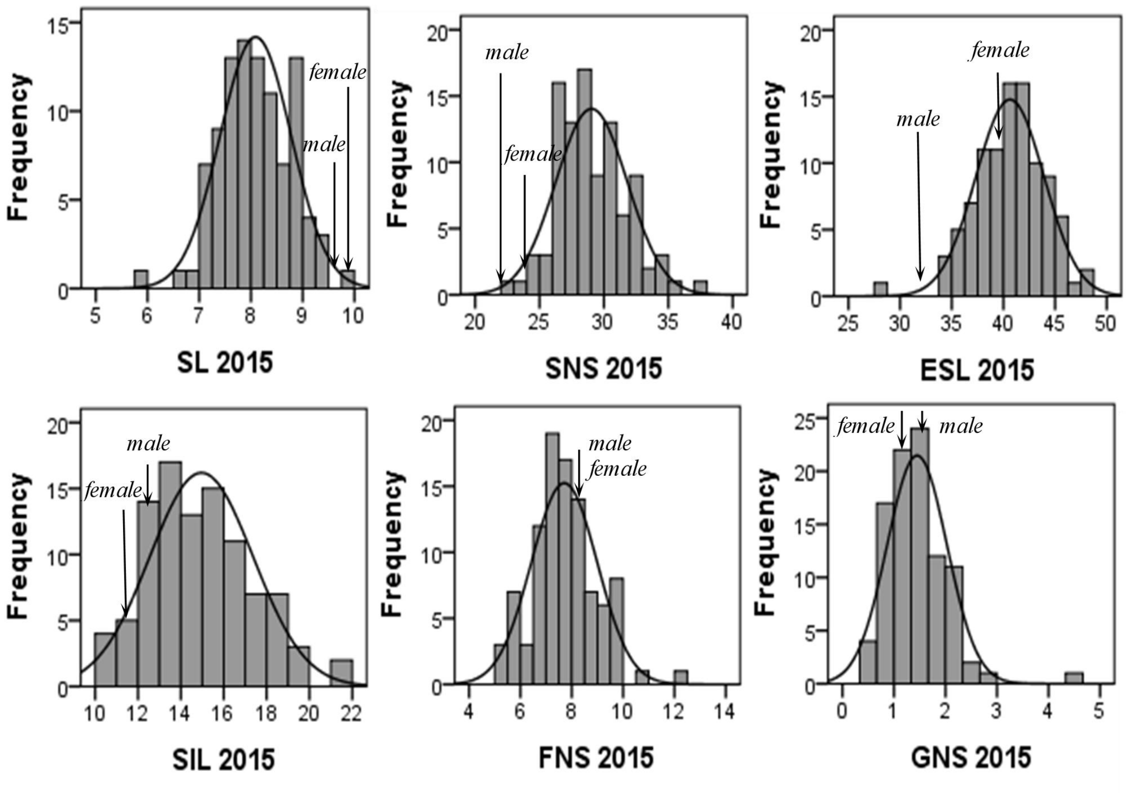

FIGURE B Phenotypic analysis of six traits of 3 environments in 2015.

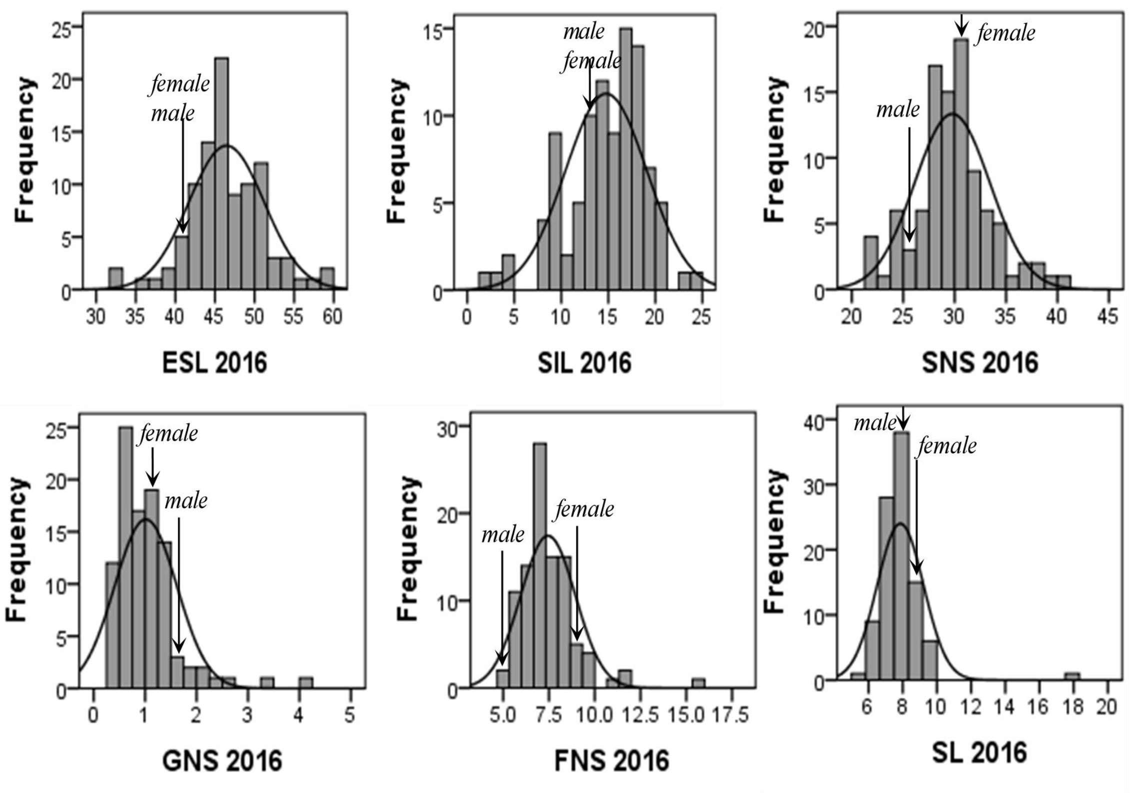

FIGURE C Phenotypic analysis of six traits of 3 environments in 2016.
